# Detection of Movement and Lead-Popping Artifacts in Polysomnography EEG Data

**DOI:** 10.3390/signals5040038

**Published:** 2024-10-22

**Authors:** Nishanth Anandanadarajah, Amlan Talukder, Deryck Yeung, Yuanyuan Li, David M. Umbach, Zheng Fan, Leping Li

**Affiliations:** 1 Biostatistics and Computational Biology Branch, National Institute of Environmental Health Sciences, Research Triangle Park, NC 27709, USA; 2 Division of Sleep Medicine, Department of Neurology, University of North Carolina at Chapel Hill, Chapel Hill, NC 27514, USA

**Keywords:** artifact, EEG, polysomnography, correlation, movement, lead popping

## Abstract

Polysomnography (PSG) measures brain activity during sleep via electroencephalography (EEG) using six leads. Artifacts caused by movement or loose leads distort EEG measurements. We developed a method to automatically identify such artifacts in a PSG EEG trace. After preprocessing, we extracted power levels at frequencies of 0.5–32.5 Hz with multitaper spectral analysis using 4 s windows with 3 s overlap. For each resulting 1 s segment, we computed segment-specific correlations between power levels for all pairs of leads. We then averaged all pairwise correlation coefficients involving each lead, creating a time series of segment-specific average correlations for each lead. Our algorithm scans each averaged time series separately for “bad” segments using a local moving window. In a second pass, any segment whose averaged correlation is less than a global threshold among all remaining good segments is declared an outlier. We mark all segments between two outlier segments fewer than 300 s apart as artifact regions. This process is repeated, removing a channel with excessive outliers in each iteration. We compared artifact regions discovered by our algorithm to expert-assessed ground truth, achieving sensitivity and specificity of 80% and 91%, respectively. Our algorithm is an open-source tool, either as a Python package or a Docker.

## 1. Introduction

Electroencephalography (EEG) measures the electrical activity of the brain by attaching electrode leads to the surface of the scalp. Electrical signals generated in the brain travel through the skull and are channeled by leads to electronic recording devices. The electric potentials of EEG signals are low, measured in micro volts (μV), and subject to distortion caused by extraneous signals. Such EEG artifacts can come from other intrinsic/physiological activities within the body or extrinsic/non-physiological electric signals from the environment [1,2]. Physiological artifacts include electric signals generated from organs or muscles associated with pumping blood, breathing, or eye movements. Non-physiological artifacts include signals from electric currents in nearby electric circuits and power lines. EEG artifacts can also arise when the recoding electrode leads become loose or detached (often referred to as “popped”), perhaps from head movements. Depending on their source, artifacts may occur in only one channel or in several channels simultaneously, and they may distort an EEG signal over a brief or prolonged period. Loose leads tend to result in artifacts that last from seconds to minutes and to be present in individual EEG channels.

Although physiological artifacts are largely unavoidable, researchers have developed methods to identify and/or minimize their effects (for reviews, see [1–3]). Notable approaches include independent component analysis (ICA) [4,5], canonical correlation analysis [6], and empirical mode decomposition [7].

Most artifact detection methods were designed for high-density routine EEGs that use 21–128 scalp electrodes to monitor subjects for 20–120 min; however, methods designed for routine EEGs may not always be suitable for overnight polysomnography (PSG) EEGs. PSG EEGs monitor subjects during an overnight sleep of, typically, 7–8 h but use a lower density of scalp electrodes—typically six channels. The longer duration during sleep opens a PSG EEG to additional physiological artifacts not seen in routine EEGs, such as those from bruxism and chewing. In addition, methods for outlier removal in routine EEGs that assume stationary brain activity may not work for PSG EEGs because sleep cycles render brain-wave activity during sleep inherently non-stationary.

Among the artifact detection methods specifically designed for PSG EEG [8–16], several subdivide the time axis into epochs, each of which contains multiple readings, and use epoch-specific measures of correlation [10,11,13,14]. Durson and colleagues [10,11] proposed the elimination of eye movement artifacts based, in part, on the correlation of EEG potentials with electro-oculogram (EOG) potentials, with epochs with high correlations evidencing contaminated signals. Garbaldi et al. [13] similarly used correlation between electromyography (EMG) potentials and EEG potentials and between EOG and EEG potentials to detect EMG- or EOG-related artifacts in EEG signals. Although these methods were designed to detect epoch-specific EOG- or EMG-induced artifacts, they do not explicitly address specific channels.

Only three works in the literature with respect to PSG EEG methods [14–16] explicitly state how their proposed method can identify both channel-specific and epoch-level artifacts. Of these three methods, some base artifact detection on the time sequence of EEG potentials, while others process the sequence of potentials into a spectrogram that represents the EEGs as epoch-specific power spectra covering a range of frequency bands, then use both potentials and power spectra.

Ktonas et al. [16] proposed a two-step method for artifact detection applied to 30-s epochs containing 128 samples/s of the EEG potential. The first step removes epochs containing high-amplitude EEG potentials. The second step probes whether the distribution of potentials within each remaining epoch approximates a Gaussian distribution using a chi-square goodness-of-fit statistic with a heuristically determined cutoff. The procedure removes any epochs declared non-Gaussian as artifacts.

Durka et al. [14] developed procedures to identify specific artifacts, such as those from eye movement, breathing, muscle movement, or a popped electrode. They estimated different functions of a spectrogram, each focusing on one type of artifact. For example, to detect eyeblink artifacts in 1.5 s epochs, the function that the authors chose is pairwise correlation in EEG power between certain pairs of leads; they declared epochs to contain artifacts when the correlation was below a heuristic threshold. For other types of artifacts, the authors defined the relevant threshold either heuristically or statistically.

Although designed for high-density, routine EEGs, FASTER (Fully Automated Statistical Thresholding for EEG artifact Rejection) [17] employs correlations in an interesting way for the detection of artifactual channels [17]. The algorithm identifies artifacts, epoch by epoch and channel by channel, using five steps. In one of the steps, FASTER uses the mean of the channel’s correlation coefficients with other channels as one of several criteria to identify artifactual channels. The premise is that signals (e.g., total EEG power) from neighboring channels should be highly correlated, and deviation from the presumed high correlation is indicative of the presence of outlier channels [17].

Wallant et al. [15] followed a complex multi-module strategy promoted by Nolan et al. [17] for the detection of artifacts in routine EEGs; however, Wallant et al. used different component modules because their focus was on PSG EEGs. In their method, after preprocessing, a first module screens for channels that are unusually flat or unusually noisy within 30 s epochs, a second module extracts features of the power spectrum, and the final module focuses on three types of artifacts—popping, movement, and arousal—within 1 s epochs. For the popping artifacts, the authors employed an approach that looks for rapid changes of large amplitude in potential [14]. To detect movement and arousal artifacts, the authors used an approach, like that proposed by Brunner [9] that focuses on power in the beta band (16–30 Hz) with an adaptive threshold.

Normal sleep includes cycles with alternating periods of non-rapid eye movement (NREM) and rapid eye movement (REM) sleep. None of the above-mentioned approaches appear to explicitly consider sleep cycles when using correlation between channels to detect sleep-stage-specific artifacts—at least, in part, because their goal is to remove artifacts as a preparation for automated sleep-stage classification [10,11,13]. Moreover, none of them unifies the correlation-based approaches for both channel- and epoch-specific artifacts. Instead, a correlation-based approach is considered either for the identification of epochs containing a specific type of artifact or for the detection of problematic channels.

In this study, we propose an iterative procedure to identify artifacts in PSG EEG signals from individual channels. Based on the premise that signals from neighboring channels should be highly correlated [10,11,13,17], we reasoned that the EEG power spectra at each time stamp should also be highly correlated between channels and that a pattern of low correlations suggests that lead popping or movement artifacts may have occurred during a given period. The algorithm uses the spectrogram for every channel to create a time series of average correlations between power spectra for each channel. Segments of time where a channel’s average correlation lies below data-based thresholds are declared artifacts. This manuscript describes the algorithm in detail. We compared its performance in identifying artifacts with artifacts detected by an expert using the overnight EEG trace from a real PSG study. We close with a discussion of the benefits and the shortcomings of our algorithm.

## 2. Methods

### 2.1. Algorithm

#### 2.1.1. Overview

**Figure 1.**
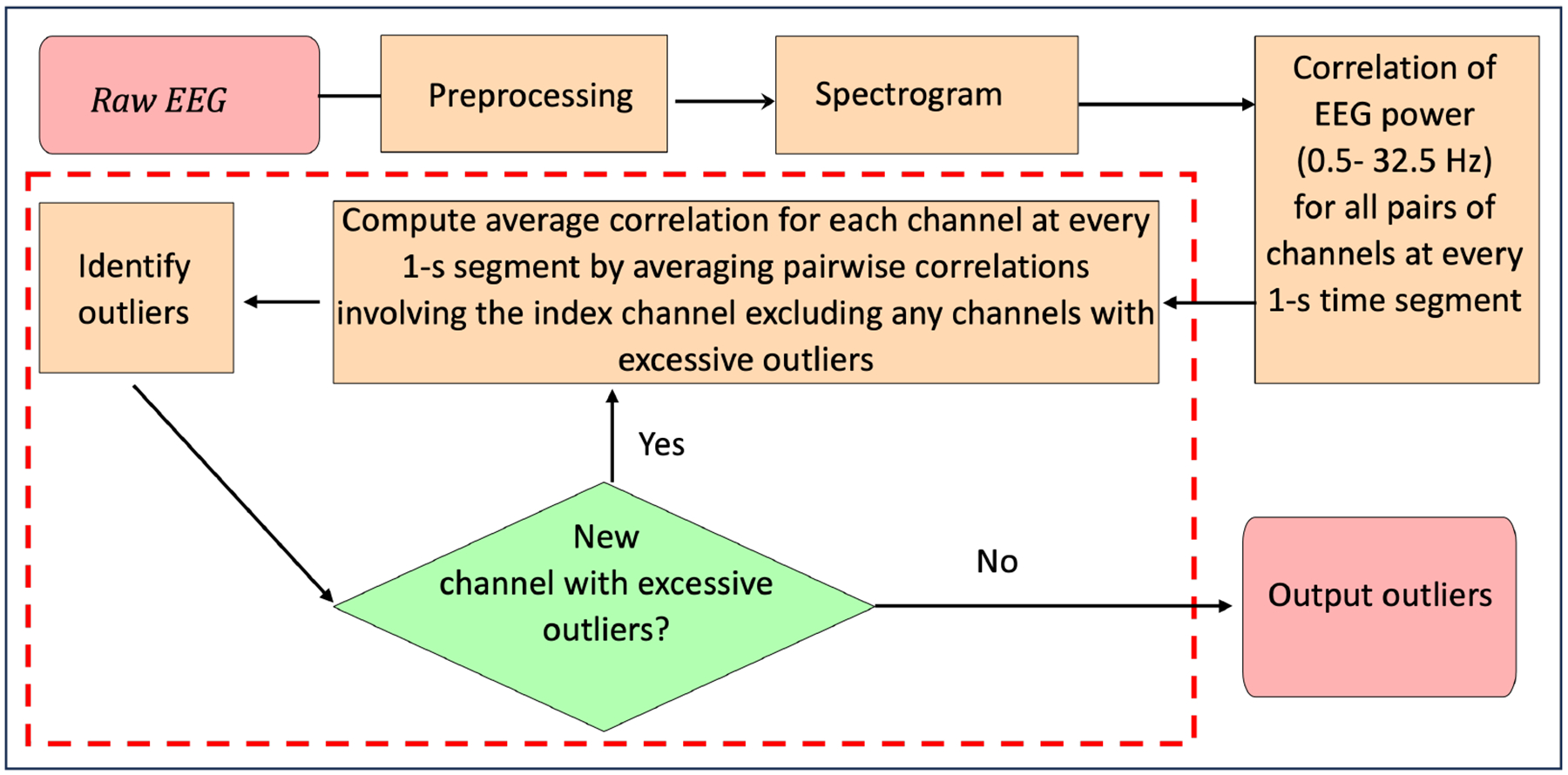
Overall workflow of the proposed method. For raw EEG data, the algorithm first performs the preprocessing step. Next, the algorithm computes pairwise correlation coefficients in EEG power between every pair of channels. For each channel, the correlations with the other channels are averaged. Based on the correlation coefficients, the algorithm iteratively identifies channels with excessive outliers and recomputes the average correlation. Finally, outlier segments are identified and annotated.

First, our algorithm (Figure 1) preprocesses the EEG potential signal to prepare for later analyses [1,3]. Next, it applies multitaper analysis to the signal from each channel (4 s intervals with 3 s overlap) to create a spectrogram that provides, for each 1 s segment, a vector of power values, with one value corresponding to each 0.25 Hz frequency band between 0.5 and 32.5 Hz. It computes the Spearman correlation coefficient between the power vectors for each 1 s segment for every pair of channels, yielding 15 time series of correlations in power—one time series for each pair of channels. From these 15 pair-specific time series, the algorithm creates six channel-specific time series of average correlations by averaging the five pairwise correlations that share a common channel segment by segment. In each channel-specific average time series, the algorithm identifies “bad” segments using a moving window-based local threshold approach [9] and considers all remaining segments “good” segments. A channel-specific global threshold for outlier detection is derived using the pool of “good” segments for that channel.

In a second pass, a 1 s segment within a channel is declared an outlier using a global threshold based on the pool of all “good” segments for the channel. Any segments falling between two outliers that are within 5 min (10 epochs) of each other are also declared outliers. Because the average correlation between any given channel and each of the other five channels can be affected by outliers in any one of the five, the algorithm proceeds iteratively. A channel with more than 5% of its segments declared as outliers is excluded from subsequent channel-specific averages of pairwise correlations but is not declared an outlier channel yet. The process is repeated until no additional channels can be excluded (Figure 1).

#### 2.1.2. Step-by-Step Description

##### Preprocessing

Our data preprocessing procedure is designed to make the input time series of potentials amenable to subsequent analyses. The algorithm uses EEG signals from six channels (F3, F4, C3, C4, O1, and O2) and preprocesses each one separately. First, it identifies 30 s epochs labeled as “Nan” or “inf” and recodes them as “NaN”, thereby making them irrelevant for downstream analysis. Each channel is re-referenced with respect to its opposite mastoid channel (M1 or M2) by subtraction, e.g., F3-M2 and F4-M1. The algorithm applies a zero-phase notch filter to remove power-line noise (60 Hz). Then the algorithm applies a band-pass filter to keep signals within the frequency range of 0.5–32.5Hz. It removes 30 s epochs with excessive flat signals, that is, epochs with amplitudes one standard deviation less than 1 μV or less than 0.2 μV for five consecutive second. To minimize movement artifacts, it also removes epochs with signal amplitudes exceeding 500 μV.

##### Spectral Analysis

Next, we carried out multitaper spectrogram (MT-spectrum) analysis [18] on the preprocessed EEG signals. For each channel, we extracted the EEG power spectrum with seven Slepian tapers using a moving window of 4 s with 3 s overlap; we indexed the period corresponding to each 4 s spectrum, termed a “segment,” to its initial second. Consequently, a segment is 1 s in duration. The spectrum for each segment consists of absolute EEG power in 128 bands, each with a 0.25 Hz resolution. Thus, we regard each segment as having a vector of power values with 128 elements.

##### Segment-Specific Correlation between Pairs of Channels

For a given pair of channels, we have two vectors of EEG power for each segment, as described above. Using those vectors, we compute the Spearman correlation for each segment of the sleep period, thereby creating a time series of correlations for the pair of channels. We create a corresponding pairwise correlation time series for all 15 possible pairs from the six channels.

Each of the six channels is paired with five other channels. We average the five correlations, segment by segment, to create a time series of average correlations for each index channel. For example, for index channel C3, we averaged all correlations involving C3, i.e., correlations between C3-C4, C3-F3, C3-F4, C3-O1, and C3-O2.

In subsequent iterations, pairwise correlations that involve a channel that subsequently recognized as having an excessive number of outlying segments (see below for definition of artifact channel) are excluded from the calculation of average correlations. Thus, after the first iteration, the average correlation for a given channel may involve fewer than five pairwise correlations.

Identification of “good” and “bad” segments. For a given channel, the algorithm characterizes each segment as “good” or “bad” using a local moving window-based approach applied to the channel’s average correlation time series. We chose a window size of 60 s (two 30 s epochs) and a sliding time of 1 s. Following Brunner et al. [9], we employed a locally adaptive threshold. We designate a segment in the moving window as a “bad” segment when its average correlation is less than half of the median of the average correlation for all segments in the window. Each segment declared “bad” is placed in the “bad” segment pool. All remaining segments, considered “good” segments, are placed in the “good” segment pool. Accordingly, each channel has its own two pools; “good” segments constitute one pool, and “bad” segments constitute the other.

Scanning each channel for outliers. Segments declared “bad” are immediately designated as outliers. To detect outlier segments among the pool of “good” segments for each channel, we adapted a global threshold approach. We derived the global threshold based on the distribution of average correlation among the “good” segments. Specifically, we considered one-fourth of the 75th percentile value of the correlations in the “good” segment pool as the threshold. Using this cutoff, we rescanned the “good” segment pool from each channel for outliers. A “good” segment was considered an outlier when its average correlation was below the cutoff. As a result, each segment in a channel was labeled as “1” (outlier) or “0” (not outlier).

Artifact regions and outlier channel(s). If the distance between two outliers (a and b) is less than 5 min (300 segments, 10 epochs), all segments between a and b are marked as outliers, creating an artifact region. If the total length of artifact regions in a channel exceeds 5% of the channel’s length after preprocessing, the channel is excluded in the next round of average correlation calculation; nevertheless, the channel is not considered an outlier channel at this stage.

#### 2.1.3. Iteration

The above steps, starting from the calculation of the average correlation for each channel to the detection of artifact regions and outlier channels, is iteratively repeated until no additional outlier channels are detected. The algorithm can be applied to the entire EEG record without regard to sleep stage or it can be applied to signals from the REM and NREM sleep stages separately.

#### 2.1.4. Data

We downloaded de-identified EEG data from 9641 in-laboratory PSG studies carried out between January 2019 and March 2023 in an American Academy of Sleep Medicine (AASM)-accredited sleep laboratory at the University of North Carolina at Chapel Hill. Each study included an electroencephalogram with at least six channels (frontal, F3 and F4; central, C3 and C4; occipital, O1 and O2; reference channels, M1 and M2), two electrooculograms, submental and bilateral tibialis surface electromyograms, and an electrocardiogram. The multi-channel polysomnograms were recorded digitally and stored using a Natus polygraph. Studies were manually scored by American Association for Sleep Medicine Board (AASM)-certified sleep technicians following guidelines from the AASM scoring manuals and interpreted by physicians certified by the American Board of Sleep Medicine. We extracted the six-channel EEG data and their corresponding sleep and wake stages (N3, N2, N1, R, and W) from the EDF files using the Python MNE package [19]. Use of these data was approved by the Institutional Review Board of the University of North Carolina at Chapel Hill (UNC-CH) (IRB #21-1984).

#### 2.1.5. Expert-Assessed Ground Truth

We demonstrated our algorithm’s performance using a single PSG EEG recording, for which we established a ground truth based on another expert’s re-examination of the de-identified EEG data. This recording was obtained in 2021 from a 19-year-old female subject. For the PSG EEG recording used for comparison, the artifacts in the PSG EEG from one of the six channels (C3) were manually annotated by an AASM-certified sleep physician. We considered the expert-annotated signal the ground truth for evaluation of algorithm performance. All subsequent discussions on performances are related this specific subject.

The expert did not attempt to annotate the entire recording at a 1 s resolution; instead, the expert identified ranges for all movement-related and loose-lead-related artifacts. Since the expert denoted the start and end positions of each artifact region, we labeled all segments from the start to the end of the artifact region as expert-identified artifacts. We counted the number of segments out the total of 46,422 that agreed in annotations between the expert and the algorithm as belonging to artifact regions. In calculating the comparison, we considered two thresholds for the local moving-window cutoff and two thresholds for the global cutoff. The comparison was restricted to NREM and REM epochs; it excluded arousal epochs.

## 3. Results

### 3.1. Channels with an Excessive Proportion of Outliers

**Figure 2.**
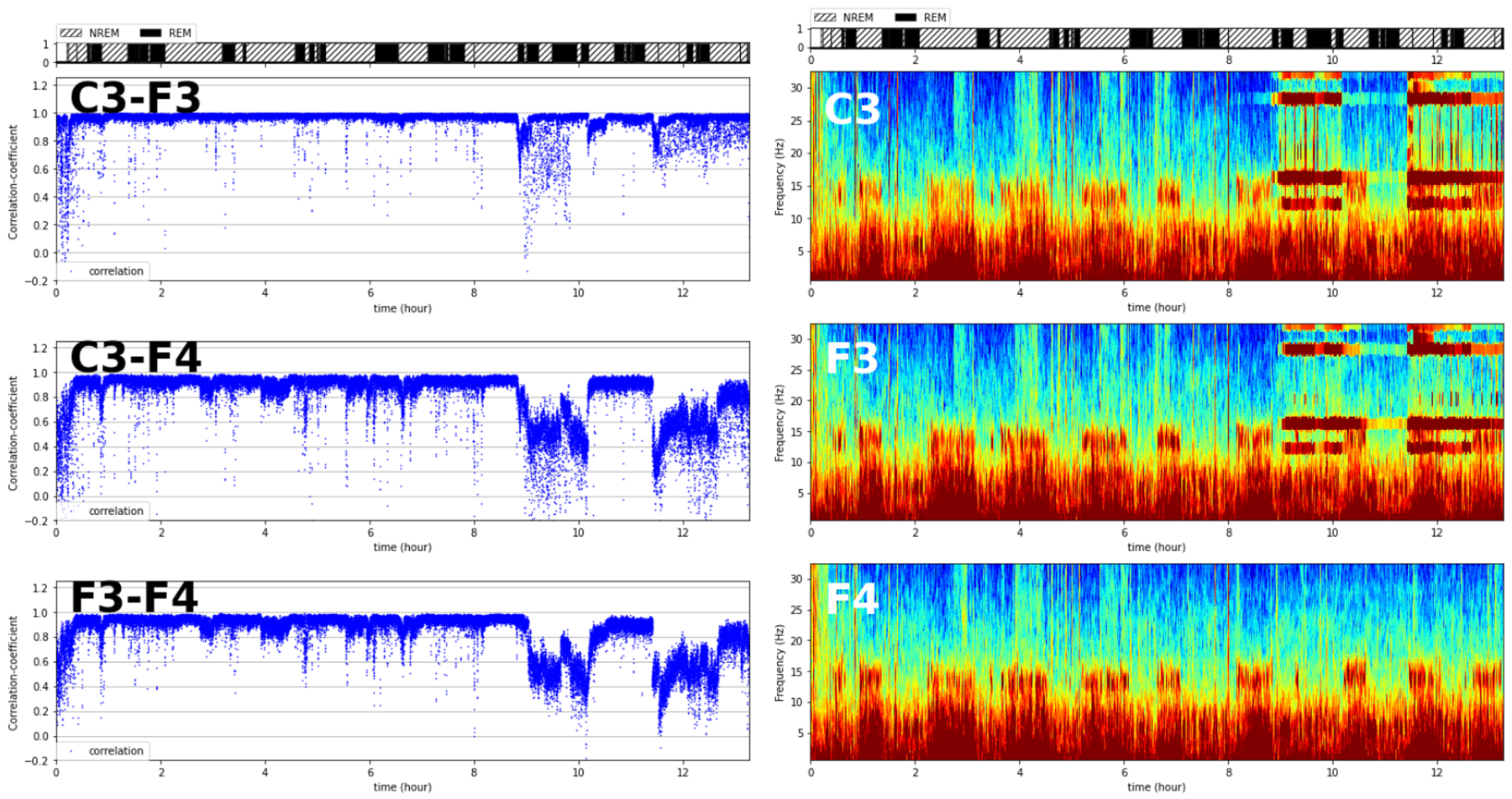
An illustration of an electrode lead popped during recording. The left panel shows the three pairwise correlations among channels C3, F3, and F4 plotted against sleep time for a single subject. Each blue dot represents the Spearman correlation coefficient between the EEG power levels in two channels in one segment. The right panel shows the overnight spectrograms for the three channels. Colors from cool to warm indicate low to high power, respectively.

**Figure A1.**
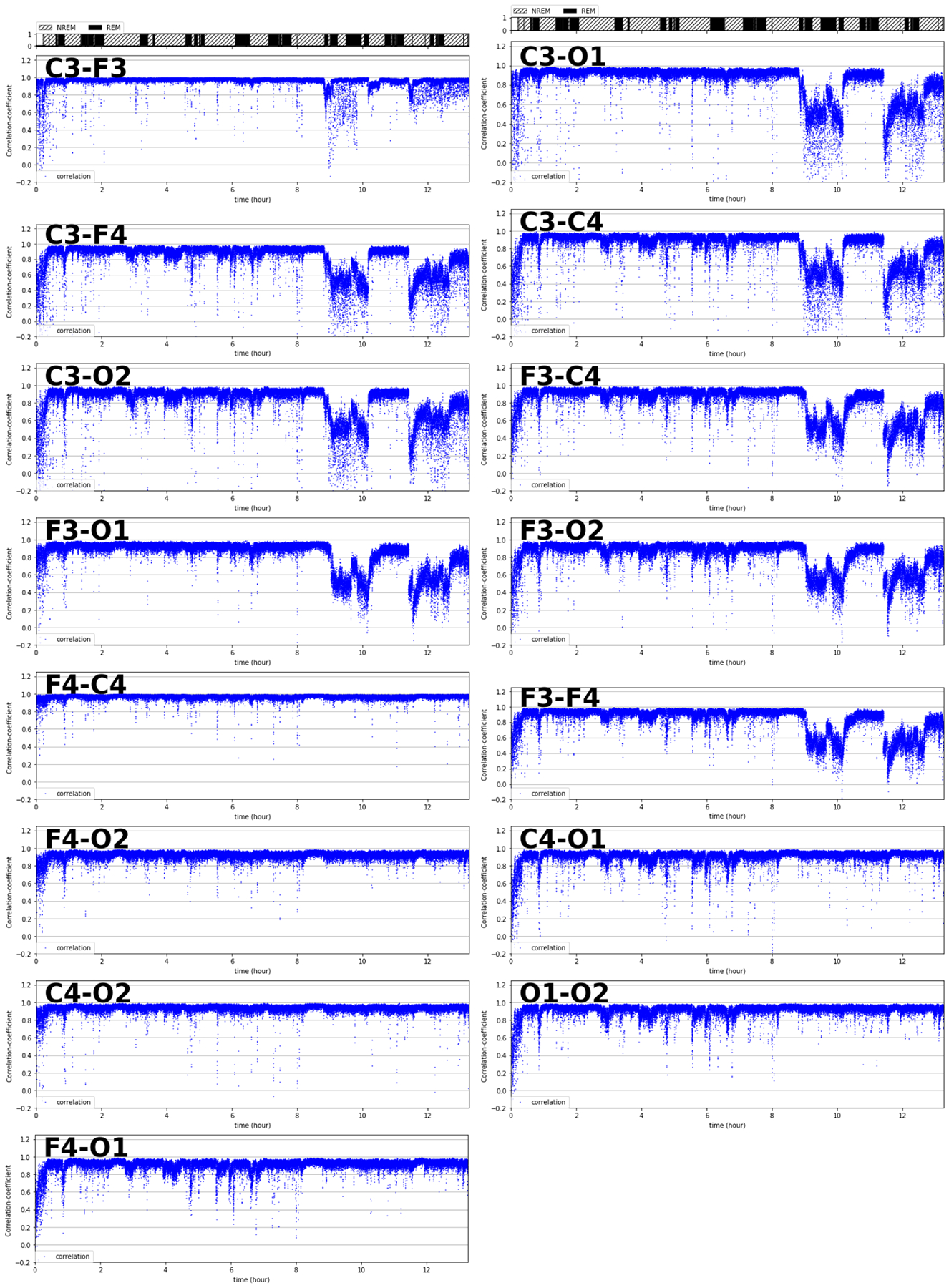
Time-series plots of correlation coefficients for all 15 possible pairs of the six channels for one subject. Each blue dot represents the Spearman correlation coefficient between the EEG power levels in two channels at one 1 s time segment. Bars at the top of each column indicate the sleep stage (NREM or REM).

**Figure A2.**
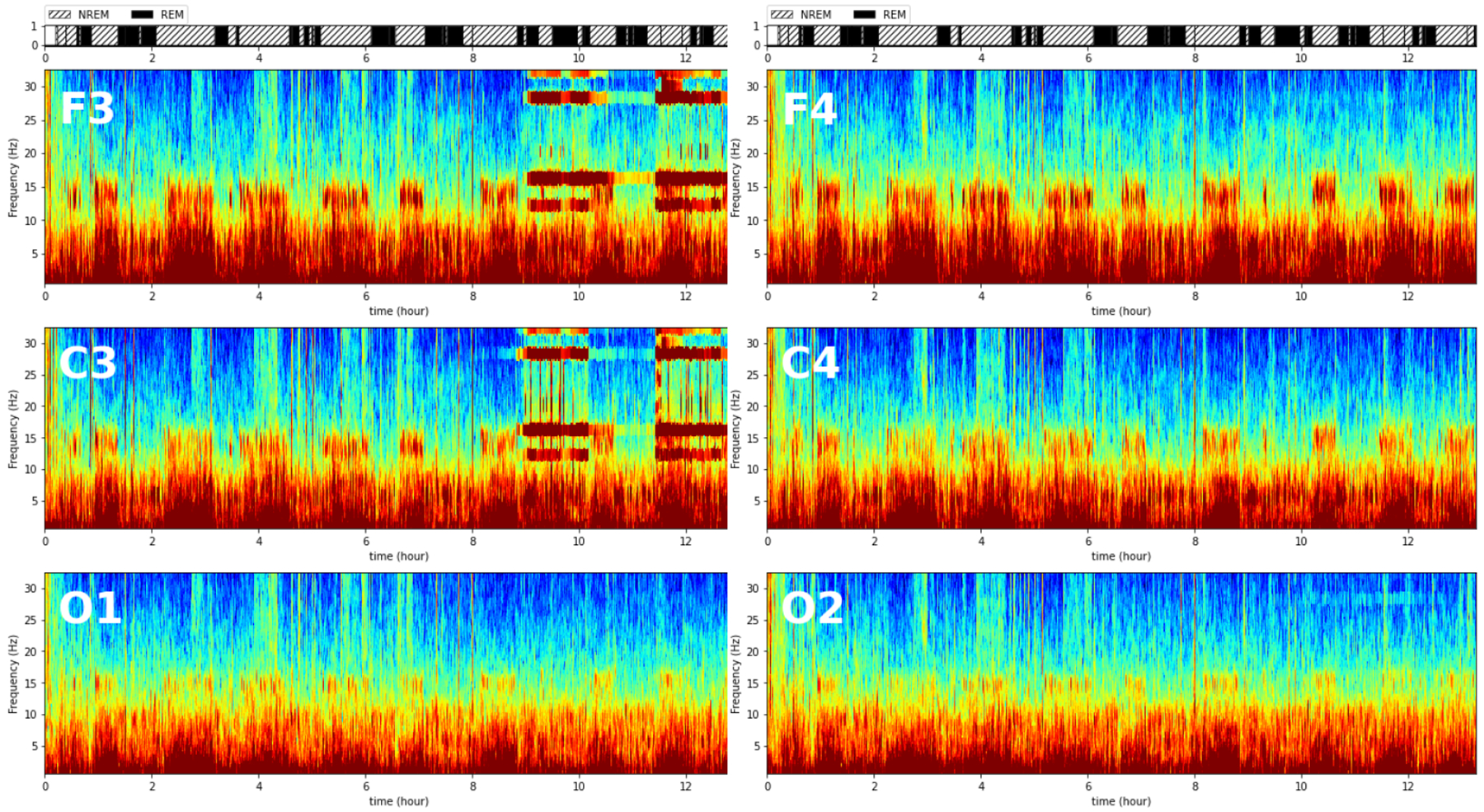
Spectrograms for each of the six channels for one subject, together with graphical depictions of sleep stage (NREM vs. REM or, more finely, Wake, REM, N1, N2, and N3).

Among 15 correlation time-series correlation plots that compare channels pairwise, the nine time-series plots for pairs where either C3 or F3 is one of channels involved show a noticeably different pattern relative to the remaining six plots (Figure A1), suggesting that channels C3 and F3 somehow behave differently from other channels, especially later in the sleep period, when the nine plots involving C3 or F3 exhibit a dramatic drop in correlation. Limiting the visualization to pairs among channels C3, F3, and F4 but adding a side-by-side view of their spectrograms reinforces the unusual features of C3 and F3 (Figure 2 compared with Figure A2). The spectrograms for C3 and F3 are similar but distinct from that for F4 and show extended stretches of unusual EEG signal beginning around 9 h of sleep. We interpret these patterns as indicating that the electrodes for the two left-hemisphere channels (C3 and F3) may be popped during this period.

**Figure 3.**
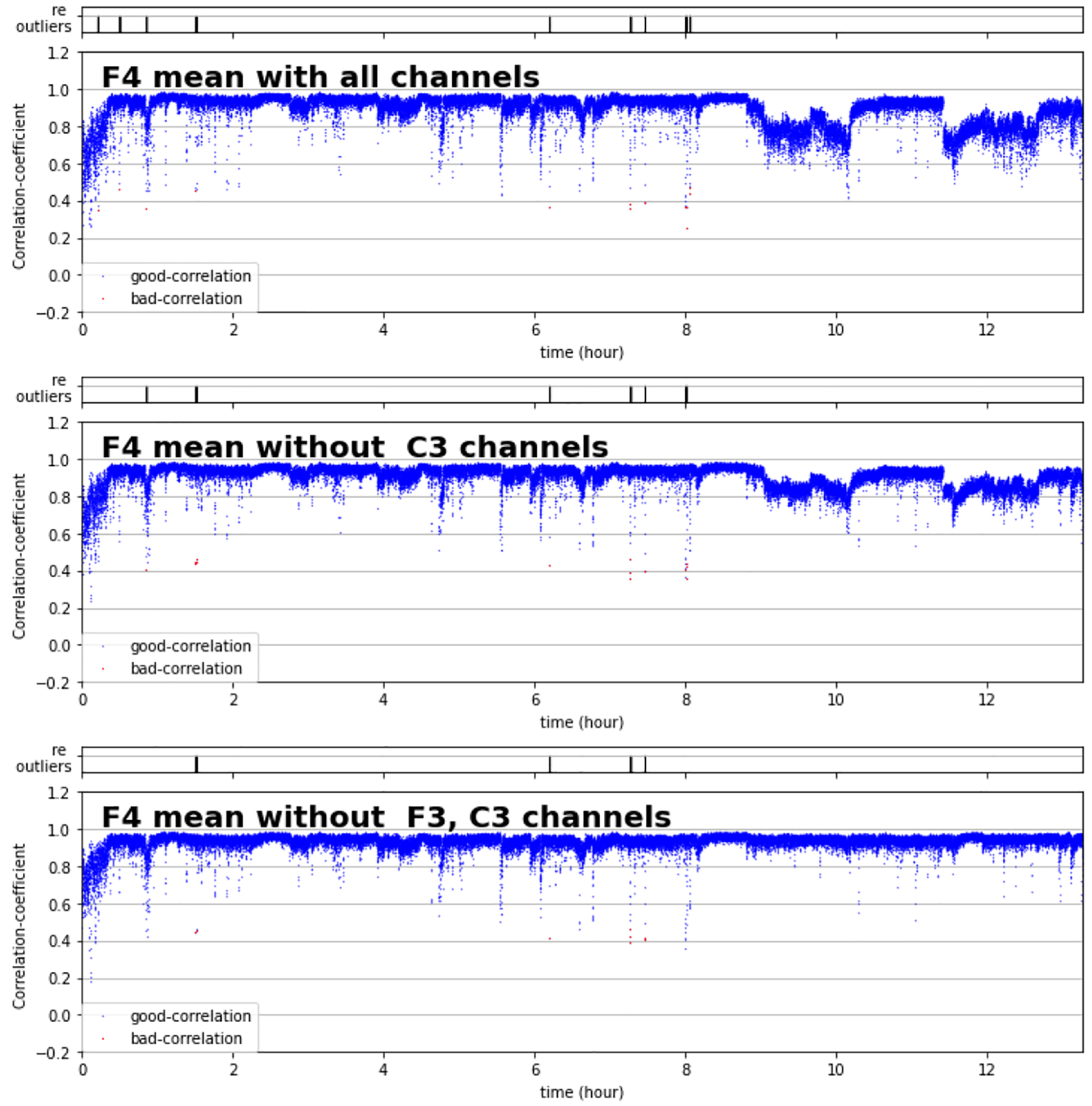
An illustration of the effect of sequentially eliminating channels found to have an excessive number of outlier segments on the average correlation time series for a given channel. Each panel represents a time series of average correlations for channel F4. Top panel: average calculated from all five pairwise correlations that include channel F4. Middle panel: average calculated from four of five pairwise correlations (omitting the F4–C4 correlation). Bottom panel: average calculated from three of five pairwise correlations (omitting F4–C4 and F4–F3 correlations). Each blue dot represents the average of pairwise Spearman correlation coefficients appropriate to the particular panel.

Indeed, our algorithm first identified C3 as having an excessive proportion of outlying segments (>5%) and subsequently removed it from the calculation of average correlation. In the subsequent iteration, our algorithm identified F3 as having excessive outliers and excluded it from the next calculation of average correlation. Besides channels C3 and F3, no additional channels were identified with excessive outliers, and iteration stopped. After removing C3 and F3 from the calculation of the F4 average correlation time series, the resulting plot lacked evidence for a large bolus of segments with low correlations in the later stages of sleep (Figure 3).

### 3.2. Segment Outliers

The PSG EEG data for the test subject were 47,733 s long (47,733 segments) (≈13.3 h), among which 46,422 segments were NREM or REM sleep. With its local threshold only, the algorithm detected 93 and 703 “bad” segments for the F3 and C3 channels, respectively, but it detected only 15–78 “bad” segments in each of the other channels (Table A1).

**Figure 4.**
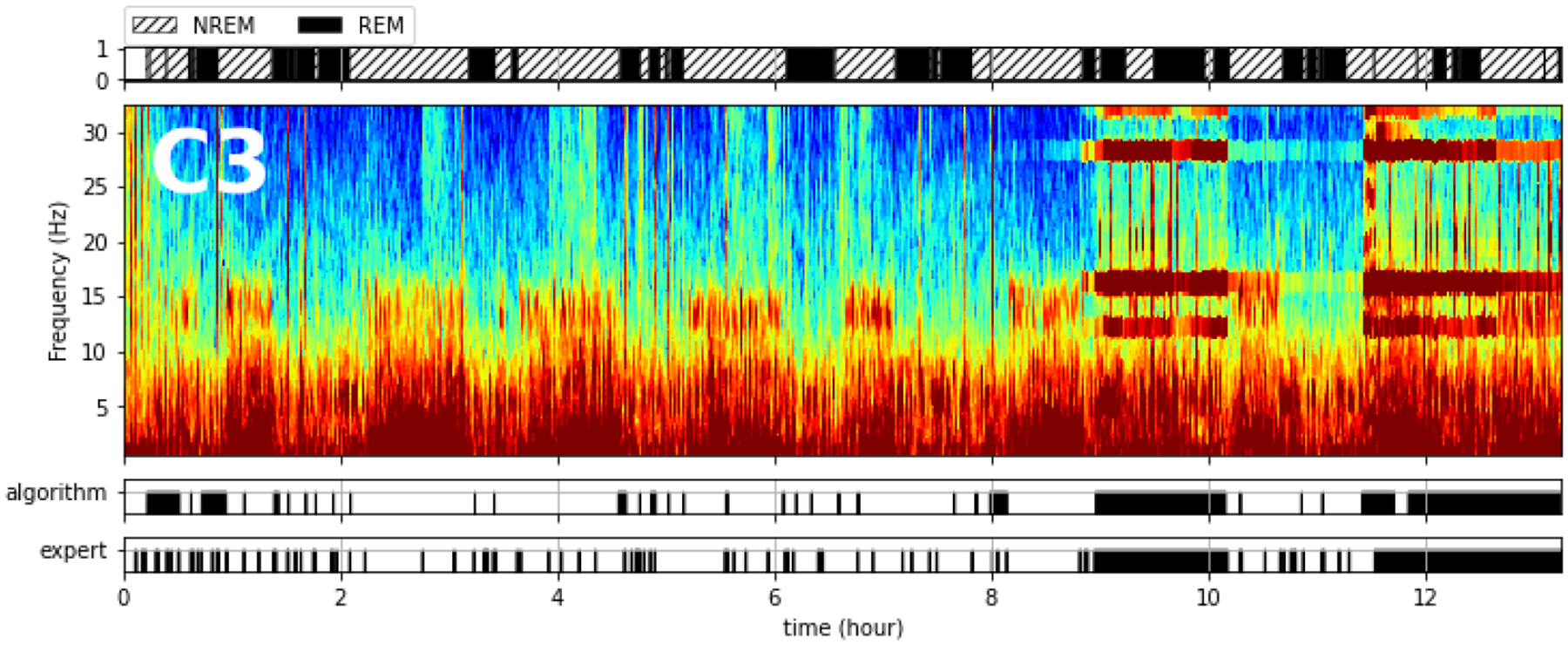
An illustration comparing expert-annotated artifact regions and algorithm-identified artifact regions in channel C3 for a single subject. Top panel: timing of NREM and REM sleep cycles. Second panel from top: spectrogram with frequency (Hz) on the Y axis, time on the X axis, and EEG power color-coded with cool to warm representing low to high power. Third panel from top: algorithm-identified artifact regions marked as black bands. Bottom panel: expert-annotated artifact regions marked as black bands.

**Figure A3.**
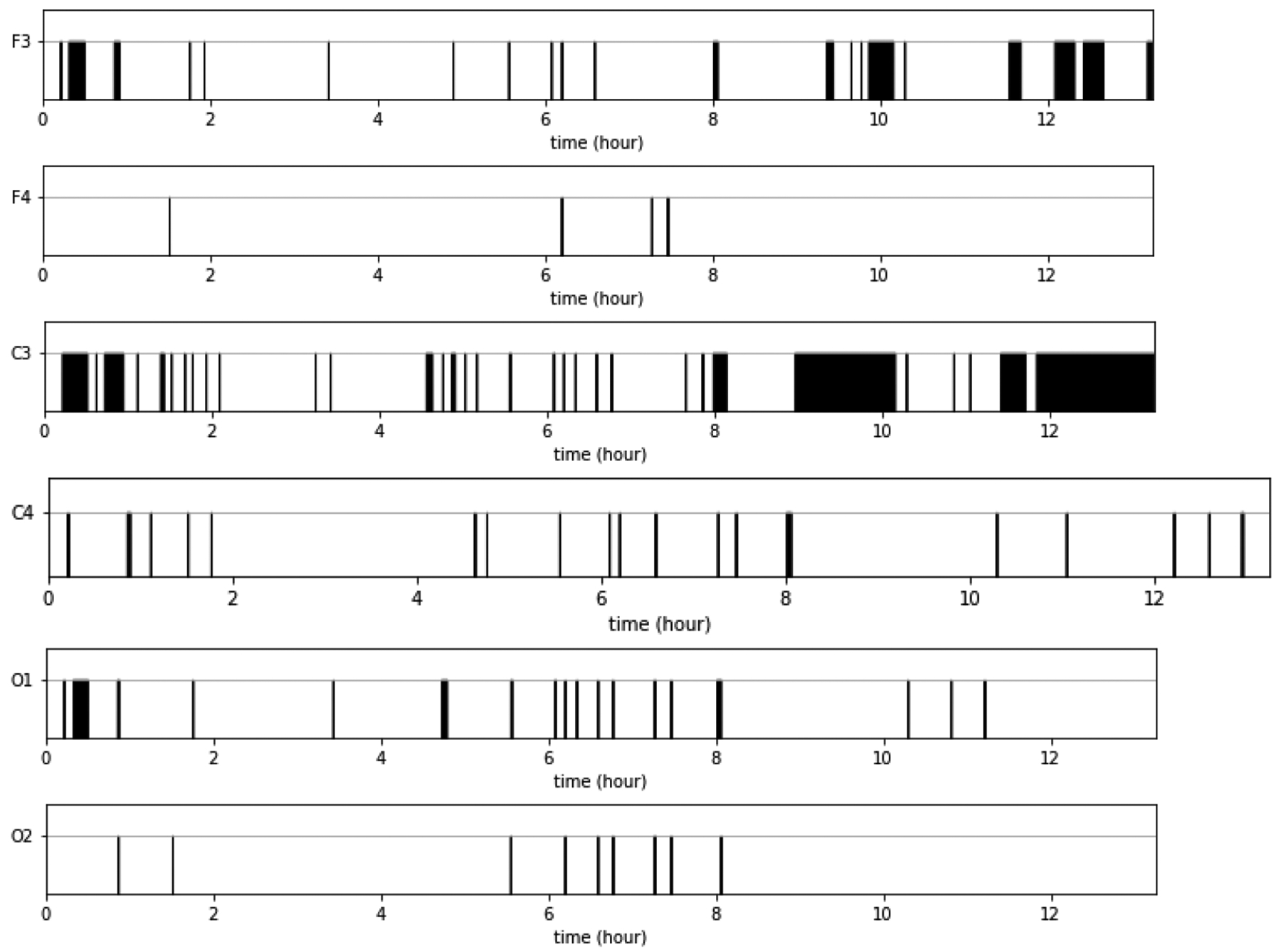
Artifact regions as annotated by the algorithm for each of the six channels. Black vertical bars mark artifact regions.

After applying the global threshold, the algorithm identified 103 and 718 outlier segments for the F3 and C3 channels, respectively (Table A2). The numbers of outliers for the other four channels remained close to the number of “bad” segments in each of those channels. Next, all segments in a region between two outliers that spanned fewer than 10 epochs (5 min) were marked as outliers. Finally, this annotation resulted in 4820 and 13,362 segments being designated as outliers for channels F3 and C3, respectively. Most outlier segments occurred during the last hour of recording (Figure 4), when the leads for the left-hemisphere recording (C3 and F3) were likely popped (Figure A3).

## 4. Comparison with the Expert-Assessed Ground Truth

The expert-assessed ground truth was based on the manual annotation of the C3 channel of the PSG EEG by an AASM-certified sleep physician. The PSG EEG had 1848 epochs. Some of the NREM and REM artifact epochs identified in the preprocessing step were excluded from the comparison. As a result, the comparison involved 46,422 segments (1562 of the 1848 total epochs) during NREM and REM sleep. We compared expert-annotated artifact regions with those identified by the algorithm by counting segments that the two classifications had in common.

The performance of the algorithm as measured by kappa and accuracy was largely insensitive to the choices of the two thresholds (Table 1). For the default choices, the overall accuracy was around 88%, with sensitivity and specificity of 80% and 91%, respectively. Systematic performance comparisons for additional local and global thresholds are presented in Tables A3 and A4.

## 5. Software Tool

We provided an open-source software tool for the algorithm. The software sets the default threshold for the local cutoff as half of the median correlation value of a moving window (default: 60 s). The default threshold for the global cutoff is one-fourth of the 75th percentile of the correlation values of all “good” segments. The choices for all parameters can be set by the user.

As for the output, the tool provides annotations for the identified artifacts in a separate file in the ascii text format. Additional capabilities of the software tool can also be found in the software package deposited on GitHub (https://github.com/nishanthCACS/EEG_PSG_loose_lead_test, accessed on accessed on 13 October 2024) and Docker (https://hub.docker.com/r/nishyanand/loose_lead_test, accessed on 13 October 2024).

## 6. Discussion

PSG EEG artifacts are unavoidable. Although some PSG EEG artifacts are detected by sleep technicians, additional artifacts are likely, despite technicians’ best efforts. Not only is manual inspection of a large number of PSG studies not feasible [16], but manual artifact annotation can be inconsistent among technicians [3].

Automated methods for EEG artifact removal can be categorized into artifact minimization and artifact identification methods [3]. Not all published works explicitly state which of these two categories the proposed method falls into. This matters because artifact minimization tries to remove contaminated signals in EEG but not epochs where contamination is present. On the other hand, artifact identification methods usually designate epochs as artifacts and subsequently eliminate those epochs entirely from further consideration in the downstream analysis. Methods for artifact minimization include those proposed in [4,5,10,11,20–22]. A notable method for artifact minimization is the ICA method [4,5], which assumes that intrinsic EEG signals and artifacts are independent components of the data signal [3]; however, if a channel is an outlier channel, i.e., with an excessive number of epochs containing artifacts, including such a channel in the ICA analysis can be problematic. Thus, it is important that channels with excessive outliers be identified first and removed before applying ICA.

A limited number of artifact identification and immunization methods for PSG EEG data have been reported [8–10,12,13]; however, those methods largely focus on identifying contaminated epochs but not on identifying artifactual EEG channels.

In this study, we developed a correlation-based approach for outlier detection. Our method is different from the existing methods in several ways. First, our method can be applied uniformly to the entire overnight sleep period or separately to NREM and REM sleep periods using sleep-period-specific local and global cutoffs. Secondly, our method uses an iterative procedure to identify and remove channels with excessive outliers to obtain an accurate representation of the channel-specific correlations with other channels. Thirdly, our streamlined method applies the same iterative procedure to all channels to identify artifacts, unlike some of the existing methods that use different procedures for different channels and different types of artifacts. Lastly, our method identifies potential outliers ranging in duration from seconds to minutes, channel by channel.

Our idea is similar to that of the FASTER algorithm [17], which was designed for high-density routine EEGs. We took a similar approach by computing the correlation in the EEG spectra between two channels at each time stamp but not the correlation between one channel and another channel across time. Specifically, we computed the correlation between the EEG power spectra in a frequency range of 0.5 Hz to 32.5 Hz at a given 1 s time segment from one channel (e.g., F3) and the EEG powers at the same frequency range at the same time segment but from another channel (e.g., C3), resulting in one correlation coefficient per time segment.

Our algorithm identified artifacts in the frequency domain (after multitaper spectrogram analysis), and the expert annotated artifacts in the time domain. Small misalignments can occur. Nonetheless, we showed that our method can effectively identify and label outlying segments and amalgamate them into artifactual regions that reflect expert annotation. Depending on their needs, users can choose to ignore individual outlying segments or very short, isolated artifactual regions.

Our paper has limitations. First, we were unable to thoroughly evaluate our method’s performance using additional expert-annotated examples. Secondly, our method may perform poorly if all channels experience artifactual regions simultaneously because of a common disturbance; then, the correlations between channels in those regions may remain nearly as high as correlations in non-artifactual regions. Any method would likely perform poorly in situations like that. However, we believe that such situations are rare.

## 7. Conclusions

Methods for artifact identification in PSG EEGs are needed. We presented an approach that is based on the premise that normal EEG power spectra at the same time should be highly correlated between any two channels, whereas a substantial reduction in correlation may be indicative of an artifact. Using this approach, we showed that individual outliers at each time segment or longer-duration artifactual regions can be identified using an iterative approach. We evaluated the performance of our method using an expert-annotated EEG recording. We showed that our method achieved a sensitivity and specificity of 80% and 91%, respectively. We also developed a software package for the tool for public use, which is deposited on GitHub (https://github.com/nishanthCACS/EEG_PSG_loose_lead_test, accessed on 13 October 2024)and Docker (https://hub.docker.com/r/nishyanand/loose_lead_test, accessed on 13 October 2024).

## Figures and Tables

**Table 1.** Comparative performance in identification of outlying segments between annotation by an expert and the use of the proposed algorithm with different thresholds.

Local^1^ Threshold	Global^2^ Threshold	Confusion Matrix				Kappa	Accuracy (%)
		TP	FP	FN	TN		
2/3	1/2	11,259	4198	1700	29,265	0.702	87.3
	1/4	11,216	4198	1743	29,265	0.699	87.2
1/2	1/2	11,112	3751	1847	29,712	0.713	87.9
(default)	1/4 (default)	10,343	3019	2616	30,444	0.701	87.9

TP: true positive; FP: false positive; FN: false negative; TN: true negative.

1 Local threshold is set at the stated fraction of the median of the average correlation for all segments in the window for the channel.

2 Global threshold is set at the stated fraction of the 75th percentile of all average correlations in the “good” segment pool for that channel.

## Data Availability

Data will be released with the written request to li3@niehs.nih.gov (L.L.).

## Appendix A

**Table A1.** Number of segments declared outliers out of 46,422 for each of the six channels using nine different tuning parameter settings with three local window sizes and three local thresholds, without invoking the global threshold.

Local Window (min)	Local^1^ Threshold	Number of Declared Outlier Segments by Channel						
		F3	F4	C3	C4	O1	O2	Total
60	2/3	353	122	1242	204	178	184	184
60	1/2	93	15	703	78	41	36	36
60	1/4	11	0	282	8	4	4	4
90	2/3	372	123	1251	207	179	189	189
90	1/2	101	16	715	77	41	38	38
90	1/4	12	0	290	8	4	4	4
180	2/3	372	129	1257	217	183	188	188
180	1/2	99	16	716	78	41	38	38
180	1/4	13	0	288	9	4	4	4

1 Thresh (threshold) is set at the stated fraction of the median of the average correlation for all segments in the window for the channel.

**Table A2.** Number of segments declared outliers out of 46,422 for each of the six channels using nine different tuning parameter settings involving three local window sizes, three local thresholds, and one global threshold.

Local Window (min)	Local^1^ Thresh	Global^2^ Thresh	Number of Declared Outlier Segments by Channel						
			F3	F4	C3	C4	O1	O2	Total
60	2/3	1/4	357	122	1249	204	178	184	184
60	1/2	1/4	103	15	718	78	41	36	36
60	1/4	1/4	40	0	448	16	4	6	6
90	2/3	1/4	372	123	1258	207	179	189	189
90	1/2	1/4	110	16	729	77	41	38	38
90	1/4	1/4	41	0	451	16	4	6	6
180	2/3	1/4	372	129	1257	217	183	188	188
180	1/2	1/4	108	16	733	78	41	38	38
180	1/4	1/4	41	0	448	16	4	6	6

1 Local thresh is set at the stated fraction of the median of the average correlation for all segments in the window for the channel.

2 Global thresh is set at the stated fraction of the 75th percentile of all average correlations in the “good” segment pool for that channel.

**Table A3.** Comparative performance in identification of artifactual regions between annotation by an expert and the use of the proposed algorithm with different local window sizes, different thresholds, and different distance cutoffs for the joining of outlier segments into an artifactual region. Performance comparisons between the expert-annotated artifacts and the algorithm-identified artifacts. Confusion matrix entries indicate the number of segments out of 46,422. The algorithm has four tuning parameters (local window size, local threshold, global threshold, and distance cutoff for the joining of individual artifacts into a continuous region of artifacts). Only a Kappa > 0.65 is presented for fixed local window sizes 60 s and 90 s.

Local Window (min)	Local^1^ Thresh	Global^2^ Thresh	Distance b-a (min)	Confusion Matrix				Kappa	Accuracy (%)
				TP	FP	FN	TN		
60	2/3	1/2	5	11,259	4198	1700	29,265	0.702	87.3
	2/3	1/2	6	11,470	5590	1489	27,873	0.655	84.8
	1/2	1/2	4	11,429	5590	1530	27,873	0.652	84.7
	1/2	1/2	5	11,112	3751	1847	29,712	0.713	87.9
	1/2	1/2	6	11,268	4509	1691	28,954	0.689	86.6
	1/4	1/2	5	11,112	3751	1847	29,712	0.713	87.9
	1/4	1/2	6	11,268	4509	1691	28,954	0.689	86.6
	2/3	1/4	4	11,470	5590	1489	27,873	0.655	84.8
	2/3	1/4	5	11,216	4198	1743	29,265	0.699	87.2
	2/3	1/4	6	11,429	5590	1530	27,873	0.652	84.7
	1/2	1/4	4	11,268	4509	1691	28,954	0.689	86.6
	1/2	1/4	5	10,343	3019	2616	30,444	0.701	87.9
	1/2	1/4	6	10,343	3019	2616	30,444	0.701	87.9
	1/4	1/4	4	11,268	4509	1691	28,954	0.689	86.6
	2/3	1/10	4	11,429	5590	1530	27,873	0.652	84.7
	2/3	1/10	5	11,216	4198	1743	29,265	0.699	87.2
	2/3	1/10	6	11,429	5590	1530	27,873	0.652	84.7
	1/2	1/10	5	10,343	3019	2616	30,444	0.701	87.9
	1/2	1/10	6	10,452	3838	2507	29,625	0.671	86.3
90	2/3	1/2	6	11,470	5588	1489	27,875	0.655	84.8
	1/2	1/2	4	11,429	5588	1530	27,875	0.652	84.7
	1/2	1/2	5	11,429	5588	1530	27,875	0.652	84.7
	1/2	1/2	6	11,268	4510	1691	28,953	0.689	86.6
	1/4	1/2	4	10,452	3839	2507	29,624	0.671	86.3
	1/4	1/2	5	10,452	3839	2507	29,624	0.671	86.3
	1/4	1/2	6	11,268	4510	1691	28,953	0.689	86.6
	2/3	1/4	4	11,470	5588	1489	27,875	0.655	84.8
	2/3	1/4	5	11,470	5588	1489	27,875	0.655	84.8
	2/3	1/4	6	11,429	5588	1530	27,875	0.652	84.7
	1/2	1/4	4	11,268	4510	1691	28,953	0.689	86.6
	1/2	1/4	5	11,268	4510	1691	28,953	0.689	86.6
	1/2	1/4	6	10,452	3839	2507	29,624	0.671	86.3
	1/4	1/4	4	11,268	4510	1691	28,953	0.689	86.6
	1/4	1/4	5	11,268	4510	1691	28,953	0.689	86.6
	2/3	1/10	4	11,429	5588	1530	27,875	0.652	84.7
	2/3	1/10	5	11,429	5588	1530	27,875	0.652	84.7
	2/3	1/10	6	11,429	5588	1530	27,875	0.652	84.7
	1/2	1/10	4	10,452	3839	2507	29,624	0.671	86.3
	1/2	1/10	5	10,452	3839	2507	29,624	0.671	86.3
	1/2	1/10	6	10,452	3839	2507	29,624	0.671	86.3

TP: true positive; FP: false positive; FN: false negative; TN: true negative.

1 Local threshold is set at the stated fraction of the median of the average correlation for all segments in the window for the channel.

2 Global threshold is set at the stated fraction of the 75th percentile of all average correlations in the “good” segment pool for that channel.

**Table A4.** Comparative performance in identification of artifactual regions between annotation by an expert and the use of the proposed algorithm for a fixed local window size (60s), fixed local threshold, and fixed global threshold but with different distance cutoffs for the joining outlier segments into an artifactual region. Confusion matrix entries indicate the number of segments out of 46,422.

Local^1^ Thresh	Global^2^ Thresh	Distance b-a (min)	Confusion Matrix				Kappa	Accuracy (%)
			TP	FP	FN	TN		
1/2	1/4	1	11,268	4509	1691	28,954	0.689	86.6
1/2	1/4	2	11,268	4509	1691	28,954	0.689	86.6
1/2	1/4	3	11,268	4509	1691	28,954	0.689	86.6
1/2	1/4	4	11,268	4509	1691	28,954	0.689	86.6
1/2	1/4	5	10,343	3019	2616	30,444	0.701	87.9
1/2	1/4	6	10,343	3019	2616	30,444	0.701	87.9
1/2	1/4	7	10,343	3019	2616	30,444	0.701	87.9
1/2	1/4	8	11,202	6044	1757	27,419	0.621	83.2

TP: true positive; FP: false positive; FN: false negative; TN: true negative.

1 Local threshold is set at the stated fraction of the median of the average correlation for all segments in the window for the channel.

2 Global threshold is set at the stated fraction of the 75th percentile of all average correlations in the “good” segment pool for that channel.

## Abbreviations

The following abbreviations are used in this manuscript:

EEG: Electroencephalography
PSG: Polysomnography
ICA: Independent component analysis
EOG: Electro-oculogram
EMG: Electromyography
NREM: Non-rapid eye movement
REM: Rapid eye movement
AASM: American Academy of Sleep Medicine
